# Should Behavior Harmful to Others Be a Sufficient Criterion of Mental Disorders? Conceptual Problems of the Diagnoses of Antisocial Personality Disorder and Pedophilic Disorder

**DOI:** 10.3389/fpsyt.2020.558655

**Published:** 2020-09-15

**Authors:** Ricarda Münch, Henrik Walter, Sabine Müller

**Affiliations:** Research Division of Mind and Brain, Department of Psychiatry and Psychotherapy, CCM, Charité-Universitätsmedizin Berlin, Corporate Member of Freie Universität Berlin, Humboldt-Universität zu Berlin, and Berlin Institute of Health, Berlin, Germany

**Keywords:** antisocial personality disorder, psychopathy, dissocial personality disorder, pedophilic disorder, pedophilia, diagnostic criteria, definition of mental disorder, harmful behavior

## Abstract

Generally, diseases are primarily harmful to the individual herself; harm to others may or may not be a secondary effect of diseases (*e.g.*, in case of infectious diseases). This is also true for mental disorders. However, both ICD-10 and DSM-5 contain two diagnoses which are primarily defined by behavior harmful to others, namely Pedophilic Disorder and Antisocial (or Dissocial) Personality Disorder (ASPD or DPD). Both diagnoses have severe conceptual problems in the light of general definitions of mental disorder, like the definition in DSM-5 or Wakefield’s “harmful dysfunction” model. We argue that in the diagnoses of Pedophilic Disorder and ASPD the criterion of harm to the individual is substituted by the criterion of harm to others. Furthermore, the application of the criterion of dysfunction to these two diagnoses is problematic because both heavily depend on cultural and social norms. Therefore, these two diagnoses fall outside the general disease concept and even outside the general concept of mental disorders. We discuss whether diagnoses which primarily or exclusively ground on morally wrong, socially inacceptable, or criminal behavior should be eliminated from ICD and DSM. On the one side, if harming others is a sufficient criterion of a mental disorder, the “evil” is pathologized. On the other side, there are practical reasons for keeping these diagnoses: first for having an official research frame, second for organizing and financing treatment and prevention. We argue that the criteria set of Pedophilic Disorder should be reformulated in order to make it consistent with the general definition of mental disorder in DSM-5. This diagnosis should only be applicable to individuals that are distressed or impaired by it, but not solely based on behavior harmful to others. For ASPD, we conclude that the arguments for eliminating it from the diagnostic manuals overweigh the arguments for keeping it.

## Introduction

Generally, diseases are primarily harmful to the diseased individual herself either by being directly life-threatening or at least life-shortening, or by causing pain or suffering, or by impairing her ability to live in human symbiotic communities (1). Harm to others, however, may or may not be a secondary effect of diseases. A typical example are infectious diseases which harm the infected individual and possibly others as well. A mere infection, however, is not called a disease as long as it is not and will not be harmful to the infected individual herself, even if it poses a risk to others as a secondary effect. This is evident from the example of asymptomatic carriers of pathogens. Although they may transmit the pathogen to others and harm particularly vulnerable, *e.g.* immunosuppressed people, medicine does not regard them as ill.^1^ Therefore, such persons should be described as being ‘disease-causing’ for others, rather than as being ‘diseased’ themselves.

If this is true for diseases in general, that they are primarily harmful to the individual herself, it should also be true for mental disorders as long as they are viewed as a subset of diseases. This is reflected in frequently cited attempts to formulate a general definition of mental disorder, like the definition in the Diagnostic and Statistical Manual of Mental Disorders (DSM-5) (3) or the “harmful dysfunction” model by Wakefield (4). Both definitions characterize a mental disorder by, broadly speaking, a dysfunction in mental processes that is associated with harm to the affected individual.

For some psychiatric diagnoses, however, it is questionable whether the presupposition of harm to the individual really applies. We will show that several diagnoses essentially rely on behavior that is harmful to others, but not necessarily to the individual herself. This is especially true for the diagnoses “Antisocial Personality Disorder” (ASPD) in DSM-5 (or “Dissocial Personality Disorder” in ICD-10) and “Pedophilic Disorder” in DSM-5 and ICD-11.^2^ Instead, as we will show, another disease criterion comes in here: the criterion of “harm to others”.

In the case of Pedophilic Disorder, harm to others is a sufficient criterion. In the case of ASPD, it is a necessary one and, as we will argue, practically also a sufficient one. In addition to the harm criterion, getting another meaning, we will argue that the criterion of a mental dysfunction is unclear in these diagnoses. Thus, the diagnoses of ASPD and Pedophilic Disorder fall out of the general concept of diseases and even out of the general concept of mental disorders. Are they accordingly rather “moral disorders” than clinical disorders?^3^ If this is true, psychiatry contributes to a “medicalization” of morally wrong behavior (6). The conceptual problems of ASPD and Pedophilic Disorder lead to the fundamental question which criteria define a mental disorder.

The aim of this paper is to discuss whether behavior harmful to others should be a sufficient criterion of mental disorder as it is the case in the diagnoses of ASPD and Pedophilic Disorder. If we come to the conclusion that this should not be the case, the question arises whether ASPD and Pedophilic Disorder should be eliminated from the diagnostic manuals.

## Mental Disorders and Their Diagnostic Manuals

In probably no other specialty of medicine has the concept of “disease” been as contested as in psychiatry. Even though in psychiatry the term “disorder” is predominantly used, it can be regarded as synonymous to “disease”, especially regarding the practical consequences. Apart from the fundamental question whether there’s such a thing as “mental disorders” at all (7), and hence, whether psychiatry is a part of medicine at all, the nature and definition of mental disorders in general have been discussed (4, 8, 9). Other controversies concern the disorder status of specific mental conditions, the most famous example probably being the removal of homosexuality from DSM in 1973 (10, 11). A still missing stringent scientific basis and the important role of values (12) bring psychiatry into a position to constantly question its own presumptions about the concept of mental disorder.

Mental disorders are classified in two classification systems: First, the International Classification of Diseases and Related Health Problems, 10^th^ revision (ICD-10), by the World Health Organization (WHO) (13). Second, for mental disorders only, the Diagnostic and Statistical Manual of Mental Disorders, 5^th^ edition (DSM-5), published by the American Psychiatric Association (APA) (3). The latter is “viewed as representing the cutting-edge of the field” (14). Both manuals define the current state of the art in psychiatric diagnostics and thus have a huge impact on clinical use but also on public discussions about mental health and finally, through their use in forensic settings, even on court rulings. The practical implications of the diagnostic manuals thus range from the funding of treatments by the public health system to the assessment of someone’s capacity to work, and indirectly to the evaluation of diminished criminal responsibility.^4^

The diagnoses in both diagnostic manuals rely on polythetic criteria sets, of which a specified number of criteria needs to apply for a specified period of time. Since the neurobiological underpinnings and the etiology of many mental disorders are still scarcely understood, the diagnostic criteria sets consist of observable and subjective symptoms. Contrary to most cases in “somatic medicine”, there are only few additional objective tests in psychiatry to support a suspected diagnosis (*e.g.* for dementias or autoimmune encephalitis).

Given their importance in the diagnostic process, the selection and exact formulation of the criteria of mental disorders are crucial. Changes in these criteria sets have a huge impact on the prevalence of certain mental disorders and on the lives of many individuals. It is thus not surprising that every revision of the diagnostic manuals is accompanied by extended controversies about the inclusion or elimination of diagnoses and the formulation of the diagnostic criteria sets (17, 18). Frances (19), for example, sharply criticizes a “diagnostic inflation” in psychiatry which he thinks was intensified by DSM-5 by adding more diagnoses and expanding the existing ones.

## Mental Disorders Harmful to Others

The most contested diagnoses in DSM and ICD are probably the paraphilias (20) and Cluster B-personality disorders (5).^5^ Especially Pedophilic Disorder and Antisocial Personality Disorder (ASPD) or Dissocial Personality Disorder (in ICD-10) are highly controversial diagnoses. Some authors question their status as clinical disorders [for ASPD, see Charland (5)] or even their place in the manuals [for Pedophilic Disorder, see Green (22)].

Pedophilic Disorder and ASPD are particularly contested because both diagnoses are highly linked to socially deviant or even criminal behavior. Persons with ASPD and pedophilic sexual offenders have a significantly increased risk of (re-)offending (23–25). Sadler (26) calls such diagnoses “vice-laden” disorders, vice being understood in a “technical sense—as simply criminal and/or immoral thought or conduct” (p. 452) by the legal and moral standards of the respective society. The notion of “vice-ladenness” indicates that those disorders imply thoughts and behaviors typically described and assessed in moral and/or legal rather than in medical terms.

Pedophilic Disorder and ASPD are not the only mental disorders associated with behaviors usually described in moral terms and potentially harmful to others, though. A person suffering from schizophrenia, for example, will presumably show in some way socially deviant behavior and may even cause harm to others when, for example, following the commands of imperative voices. The crucial point, however, is that in the case of schizophrenia the symptoms described in the diagnostic criteria set are “relatively immune to misconstrual as vice” (6) (p. 9). Immoral or harmful behavior is not a defining criterion of the disorder, rather it may or may not be a secondary effect of it. In contrast, for ASPD and Pedophilic Disorder, behavior that is morally wrong and primarily harmful to others is a central part of the diagnosis: they are “vice-laden” at their core.

### Pedophilic Disorder

In DSM-IV, the diagnosis of pedophilia required that the fantasies, sexual urges, or behaviors involving children cause clinically significant distress or impairment in social, occupational, or other important areas of functioning (Criterion B). This criterion was changed in DSM-IV-TR so that it was then sufficient to have acted on the sexual urges. From DSM-IV-TR to DSM-5, all criteria remained unchanged after the proposed changes were declined (27, 28) (**Table 1**).^6^

**Table 1:** Comparison of the diagnostic criteria of pedophilic disorder and pedophilia in the DSM-IV, DSM-IV-TR, and DSM-5.

DSM-IV—Pedophilia (302.2)	DSM-IV-TR—Pedophilia (302.2)	DSM-5—Pedophilic Disorder (302.2)
A. Over a period of at least 6 months, recurrent, intense sexually arousing fantasies, sexual urges, or behaviors involving sexual activity with a prepubescent child or children (generally age 13 years or younger). B. The fantasies, sexual urges, or behaviors cause clinically significant distress or impairment in social, occupational, or other important areas of functioning. C. The person is at least age 16 years and at least 5 years older than the child or children in Criterion A. Note: Do not include an individual in late adolescence involved in an ongoing sexual relationship with a 12- or 13-year-old. (29)	A. Over a period of at least 6 months, recurrent, intense sexually arousing fantasies, sexual urges, or behaviors involving sexual activity with a prepubescent child or children (generally age 13 years or younger). B. *The person has acted on these sexual urges, or the sexual urges or fantasies cause marked distress or interpersonal difficulty*. C. The person is at least age 16 years and at least 5 years older than the child or children in Criterion A. Note: Do not include an individual in late adolescence involved in an ongoing sexual relationship with a 12- or 13-year-old. (30)	A. Over a period of at least 6 months, recurrent, intense sexually arousing fantasies, sexual urges, or behaviors involving sexual activity with a prepubescent child or children (generally age 13 years or younger). B. The individual has acted on these sexual urges, or the sexual urges or fantasies cause marked distress or interpersonal difficulty. C. The person is at least age 16 years and at least 5 years older than the child or children in Criterion A. Note: Do not include an individual in late adolescence involved in an ongoing sexual relationship with a 12- or 13-year-old. (3)

Text that has been changed from the previous version is shown in italics.

DSM-5, however, introduced a distinction between Pedophilia and Pedophilic Disorder. Pedophilia denotes the mere sexual preference for prepubescent children (Criterion A) and is not considered a mental disorder anymore. Pedophilic Disorder is Pedophilia with either personal distress or interpersonal difficulty, or sexual acts involving prepubescent children (Criterion B).

ICD-11, which has been presented by the WHO in 2019 and will foreseeably come into effect on 1 January 2022, adjusted the criteria of “Pedophilic Disorder” to the DSM-5 criteria (**Table 2**). Except for the time criterion (the sexual attraction to children must be present for at least 6 months), which is only required in DSM-5, the criteria in ICD-11 and DSM-5 are basically the same (**Tables 1** and **2**).

**Table 2:** Comparison of the diagnostic criteria of pedophilic disorder and pedophilia in ICD-10 and ICD-11.

ICD-10— Pedophilia (F 65.4)	ICD-11 – Pedophilic Disorder (6D32)
A sexual preference for children, usually of prepubertal or early pubertal age. Some pedophiles are attracted only to girls, others only to boys, and others again are interested in both sexes. (13)	*Pedophilic disorder is characterized by a sustained, focused, and intense pattern of sexual arousal—as manifested by persistent sexual thoughts, fantasies, urges, or behaviors—involving pre-pubertal children*. *In addition, in order for Pedophilic Disorder to be diagnosed, the individual must have acted on these thoughts, fantasies or urges or be markedly distressed by them*. *This diagnosis does not apply to sexual behaviors among pre- or post-pubertal children with peers who are close in age*. (34)

Text that has been changed from the previous version is shown in italics.

The age limit mentioned by DSM-5 (13 years) is clearly below the age of sexual consent, which ranges between 14 and 18 years in most countries (in the US states, for example, it ranges between 16 and 18 years). This means that the criterion of “has acted on these sexual urges” is equivalent to committing a criminal act.

This, however, does not apply to all countries in the world. According to the UNICEF child marriage report from 2014, about 250 million women alive today were married before age 15 (35). In some countries, this is even covered by law as it is allowed to marry before age 18 (in some cases there is no minimum age at all) under certain circumstances (36). This shows that not in every country sexual intercourse with children age 13 or younger is considered a criminal offense. Therefore, the legal and social reactions which individuals, who sexually abuse children, will have to face differ. Of course, even though tolerated by law in some countries, sexual acts involving children are harmful and should be legally forbidden all over the world.

Most researchers emphasize the difference between pedophilic interests and sexual offending against children. Not all individuals with pedophilic interests sexually approach children, and not all child molesters have “recurrent and intense” pedophilic interests; about half of the cases of sexual abuse of children are committed by presumably non-pedophilic offenders.^7^

However, both criteria A and B of Pedophilic Disorder contain a behavioral aspect that is sufficient for the respective criterion to be fulfilled. The use of the conjunction “or” before “behaviors” in criterion A makes it possible to meet this criterion solely by repeated acts of sexual behavior involving children (38). Regarding criterion B, sexual acts involving children are also sufficient to fulfill this criterion. This means that repeated sexual behavior involving children is sufficient to fulfill both criteria.

According to the criteria in DSM-5 and ICD-11, a diagnosis of Pedophilic Disorder requires neither suffering from the sexual fantasies, urges, or behaviors towards children nor experiencing any impairment in social, occupational or other important activities. The diagnosis can be made solely on grounds of behavior harmful to others. This has been criticized as a confusion of “mental disorder” and “crime” (20) or “immoral behavior” (41).

### Antisocial Personality Disorder (ASPD)

In an attempt to define reliably measurable personality traits, the DSM focused on behavior in the definition of ASPD, which was intended to be an equivalent of psychopathy (3, 42). Psychopathy, conceptualized by the Hare Psychopathy Checklist Revised (PCL-R) (24), contains much more interpersonal and affective symptoms than ASPD (25, 43) but is not a diagnosis in ICD-10 or DSM-5 (44).^8^ Almost all criteria of ASPD in DSM-5 refer to behavior primarily harmful to others (**Table 3**). In accordance with the diagnostic criteria required for all personality disorders, the antisocial personality traits must be “inflexible, maladaptive, and persistent and cause significant functional impairment or subjective distress” (3).

**Table 3:** Comparison of the diagnostic criteria of Antisocial Personality Disorder (DSM-5) and Dissocial Personality Disorder (ICD-10).

DSM-5—Antisocial Personality Disorder (301.7)	ICD-10—Dissocial Personality Disorder (F60.2)
A. A pervasive pattern of disregard for and violation of the rights of others, occurring since age 15 years, as indicated by three (or more) of the following: 1. Failure to conform to social norms with respect to lawful behaviors, as indicated by repeatedly performing acts that are ground for arrest. 2. Deceitfulness, as indicated by repeated lying, use of aliases, or conning others for personal profit or pleasure. 3. Impulsivity or failure to plan ahead. 4. Irritability and aggressiveness, as indicated by repeated physical fights or assaults. 5. Reckless disregard for safety of self or others. 6. Consistent irresponsibility, as indicated by repeated failure to sustain consistent work behavior or honor financial obligations. 7. Lack of remorse, as indicated by being indifferent to or rationalizing having hurt, mistreated or stolen from another. B. The individual is at least age 18 years. C. There is evidence of conduct disorder with onset before age 15 years. D. The occurrence of antisocial behavior is not exclusively during the course of schizophrenia or bipolar disorder.	Personality disorder, usually coming to attention because of a gross disparity between behavior and the prevailing social norms, and characterized by (at least three of the following criteria) 1. Callous unconcern for the feelings of others. 2. Gross and persistent attitude of irresponsibility and disregard for social norms, rules, and obligations. 3. Incapacity to maintain enduring relationships, though having no difficulty in establishing them. 4. Very low tolerance to frustration and a low threshold for discharge of aggression, including violence. 5. Incapacity to experience guilt, or to profit from adverse experience, particularly punishment. 6. Marked proneness to blame others, or to offer plausible rationalizations for the behavior bringing the subject into conflict with society. There may be persistent irritability as an associated feature. Conduct disorder during childhood and adolescence, though not invariably present, may further support the diagnosis.

The equivalent of ASPD in ICD-10, Dissocial Personality Disorder (DPD), refers less to behavioral and more to affective symptoms than ASPD in its criteria set (25) (**Table 3**). However, as Kröber and Lau (15) note, most of the criteria can still be “easily derived from the criminal behavior itself” (p. 681).

The general criteria of personality disorders in ICD-10 require that “the disorder leads to considerable personal distress but this may only become apparent late in its course” and “the disorder is usually, but not invariably, associated with significant problems in occupational and social performance” (13) (p. 202).^9^

However, Habermeyer states that persons with antisocial or dissocial personality traits subjectively do not suffer from their abnormalities and show little willingness to get treated (16). This is accentuated for inmates with high values on the Psychopathy Checklist (16). Many, if not the overwhelming majority of subjects with psychopathy are perfectly content with and identify with their traits; there is no subjective suffering involved in psychopathy (42). Because there is nothing painful or ego-dystonic in psychopathic symptoms, it is unlikely that a psychopath would seek or endure treatment (42). Also persons with ASPD rarely seek treatment (43, 46), indicating that they usually do not feel significantly distressed or impaired by their condition. This becomes evident from the description of the self-image of people with dissocial or antisocial personality traits by Müller-Isberner et al.: “These people generally see themselves as autonomous, strong loners. Some see themselves as exploited and mistreated by society and justify harming others by saying that they themselves are being harassed. Others see themselves as robbers in a world where the motto is ‘eat and be eaten’ or ‘the winner takes it all’ and where it is normal or even desirable and necessary to violate social rules.”^10^ (47) (p. 373).

This raises the question whether the diagnosis of ASPD could be made for anyone at all if the criteria of subjective distress and/or functional impairment were strictly applied. In clinical practice, distress can be presumed if someone seeks help voluntarily. The question is why this person seeks help and what distresses her. According to the literature on antisocial personality cited above, it is probably not her antisocial personality. However, subjective distress “in general” is not sufficient to make this specific diagnosis, even if all the other criteria of ASPD apply. According to DSM-5, the subjective distress must be caused by the antisocial personality traits.

It could be objected that a lack of personal distress in ASPD is precisely part of its psychopathology, in the sense that not recognizing one’s own problems is even more pathological than recognizing them. However, the general problem with this argument is that it allows the attribution of mental disorders to persons without personal distress from the outside. Even though there are cases in which this can be justified (*e.g.* in the case of severe psychosis/delusions where the individual doesn’t recognize her psychosis/delusions), there is a high risk of misusing psychiatric diagnoses for pathologizing socially deviant or nonconformist behavior.

The questionable personal distress in ASPD is especially relevant in the forensic context where the prevalence of ASPD is much higher than in the general population. The base rate in the population is estimated at 2%, whereas the prevalence among male prisoners is estimated at between 47 and 80% (25, 48). Prisoners are certainly distressed. However, distress because of the legally justified consequences of antisocial behavior, like a loss of freedom, must not be confused with distress because of the antisocial personality traits themselves (49). Distress because of society’s negative reaction to deviant behavior is not a sign of a mental disorder. Rather, it is normal. We suspect that the criterion of subjective distress and/or impairment often is not considered correctly when the diagnosis of ASPD is made, especially not in forensic contexts. The great difference between the prevalence of ASPD in the general population and among male prisoners indicates a strong correlation between ASPD and imprisonments. This means that either most criminals have a mental disorder or that ASPD is a construct mainly depicting criminal behavior.

We conclude that, strictly speaking, many persons diagnosed with ASPD in fact only have antisocial personality traits, which are not a mental disorder according to DSM-5. This conclusion is supported by the observation of Herpertz that a lack of considering the general definition of personality disorder and instead a focus on the easily applicable specific criteria lists led to an “inflationary diagnosis frequency” of personality disorders (50). We suspect that, especially in the case of ASPD, many persons are mistakenly classified as “mentally ill” because of a wrongful interpretation or even neglect of the distress/impairment criterion.

ICD-10 and DSM-5 present a categorial classification of personality disorders with ASPD/Dissocial Personality Disorder being a distinct disorder-entity. This categorial approach to personality disorders, however, is broadly contested (50). DSM-5 already introduced an alternative “hybrid” model for personality disorders, mixing categorial and dimensional approaches.^11^

According to the alternative model, the typical features of ASPD are “a failure to conform to lawful and ethical behavior, and an egocentric, callous lack of concern for others, accompanied by deceitfulness, irresponsibility, manipulative-ness, and/or risk taking” (p. 763). Psychopathy is described as a distinct variant that is “marked by a lack of anxiety or fear and by a bold interpersonal style that may mask maladaptive behaviors (*e.g.*, fraudulence).” (3) (p. 765).

ICD-11 goes even further in replacing the categorial model by a dimensional one (50). According to this model, the diagnosis of a personality disorder comprises three steps. First, the general criteria of a personality disorder must be met (“problems in functioning of aspects of the self […], and/or interpersonal dysfunction […] that have persisted over an extended period of time (*e.g.*, 2 years or more)”, “the disturbance is manifest in patterns of cognition, emotional experience, emotional expression, and behaviour that are maladaptive”, “the disturbance is associated with substantial distress or significant impairment in personal, family, social, educational, occupational or other important areas of functioning” (34)). Then, the severity of this general personality disorder must be determined (mild, moderate, severe). Eventually, the specific underlying personality structure is assessed according to five personality domains (negative affectivity, detachment, dissociality, disinhibition, anakastia). Thus, in ICD-11, there will be no category “Dissocial Personality Disorder” anymore. Instead, dissocial and disinhibited traits and behaviors may be a specifier among others in a diagnosis of a (general) personality disorder.

### Interim Conclusion

In both the definitions of ASPD and Pedophilic Disorder behavior harmful to others or even criminal behavior is a criterion for the diagnosis of a mental disorder. For Pedophilic Disorder, even though harming others (for a period of at least 6 months) is not a necessary criterion, it can be a sufficient one. For ASPD, repeated harming of others is a necessary criterion, and—not formally, but practically—also a sufficient one.

The key question is: Should criminal behavior/harm to others be a sufficient criterion of a mental disorder? Or does this lead to a “medicalization” of vice conditions, meaning that “all problematic deviance reflects human illness or injury, including criminality and ‘immoral’ conduct” (6) (p. 12)? The crucial point is: can behavior harmful to others alone indicate the presence of a mental disorder? Or is this rather an attempt to “pathologize the morally wrong”? We will come back to this question later.

The conceptual problems of Pedophilic Disorder and ASPD lead directly to a more fundamental question: which criteria define a mental disorder?

## The Definition of Mental Disorder

### The General Concept of Disease

If psychiatry claims to be a part of medicine, a general definition of disease should be the basis of a definition of mental disorders. Hucklenbroich developed a profound reconstruction of the general concept of disease (51). He distinguishes four levels of the concept of disease. The first level is the life-world and personal concept of disease (person X is ill). On the second level, a distinction can be made between healthy and pathological life processes (X is pathological). At the third level, reference is made to a standard model of the human organism (X is pathologically altered). At the fourth level, disease entities and categories are postulated (X is a disease). The basis of the determination of disease entities is an etiopathogenetic model that comprises an identification of primary causes and the typical clinical course.

According to this reconstruction, life processes that meet four criteria can be described as pathological: 1. They are states, processes, or procedures in individuals, 2. which are attributable to the organism, not the environment, 3. which take place independently of the will and knowledge of the affected individuals, and 4. for which there is at least one non-pathological alternative course.

To determine which processes are diseases, Hucklenbroich distinguishes positive and negative disease criteria. Positive criteria of a disease are: 1. lethality; 2. pain, discomfort, suffering; 3. disposition for 1 or 2; 4. inability to reproduce; 5. inability to live together. The two negative criteria of disease, which determine a condition as non-pathological, are 1. universal occurrence and inevitability, *e.g.* gender, intrauterine and ontogenetic phases, pregnancy, menopause, old age, natural death; 2. knowingly and intentionally self-induced behavior (as long as self-determination is not diminished), *e.g.* suicide, value judgements, risky behavior, abstinence, intentional lying.

Hucklenbroich argues that this general concept of disease also applies to mental disorders, even though an etiopathogenetic disease model like in “somatic” medicine is still missing in psychiatry (2). According to his model, especially the positive criteria 2 and 5 are relevant for mental disorders. Mental disorders are often associated with significant pain, discomfort or suffering. Additionally, they may impair the ability to live together with others in a community. However, Hucklenbroich notes that due to the lack of knowledge about the etiopathogenesis of mental disorders there are still diverging concepts of mental disorder (2).

### The DSM-5 Definition of Mental Disorder

One of the mostly cited definitions of mental disorder is given in DSM-5. While conceding that “no definition can capture all aspects of all disorders in the range contained in DSM-5” (3) (p. 20), it is stated that the definition is rather meant to formulate elements required for considering something a mental disorder:

“A mental disorder is a syndrome characterized by **clinically significant disturbance** in an individual’s cognition, emotion regulation, or behavior that reflects a **dysfunction in the psychological, biological, or developmental processes underlying mental functioning**. Mental disorders are usually associated with significant **distress or disability in social, occupational, or other important activities**. An expectable or culturally approved response to a common stressor or loss, such as the death of a loved one, is not a mental disorder. **Socially deviant behavior** (*e.g.* political, religious, or sexual) and **conflicts that are primarily between the individual and society are not mental disorders** unless the deviance or conflict results from a dysfunction in the individual, as described above.” (3) (p. 20, emphasis added)

The definition starts with 1. an observable symptom level (“clinically significant disturbance”) that is 2. caused by an underlying dysfunction in the “mental domain” of an individual, and that has 3. some expected consequences, namely distress or disability in important activities of daily life. The rest of the definition specifies circumstances under which certain conditions are not deemed mental disorders: Socially deviant behavior and conflicts between the individual and society, which are not the result of a dysfunction, are not considered mental disorders.

The last point seems to be crucial. Pedophilic Disorder and ASPD are, prima facie, conditions that are mainly based on a conflict between the individual and other individuals and/or society.^12^ A person with Pedophilic Disorder could argue that his sexual orientation simply does not fit in his society’s current concepts of approved sexual relationships while denying that sexual contacts with children are actually harmful to them.^13^ Or a person diagnosed with ASPD could argue that he does not feel bothered by his antisocial behavior because he has many advantages by it, although he might come into conflict with the law unless he is careful.

According to DSM-5, socially deviant behavior can be a sign of a mental disorder only if it results from a dysfunction in the individual’s “psychological, biological, or developmental processes underlying mental functioning”. However, the behavioral symptoms described in the diagnoses of ASPD and Pedophilic Disorder can have very different causes. Indeed, the lack of differentiation between the different causes of mental disorders is a fundamental problem of the nominalistic approach of DSM and ICD.

If hypersexual and even pedophilic behavior occurred in previously normal people after a brain tumor, a brain trauma, or epilepsy surgery, the brain pathology probably causally contributed to the abnormal behavior (54, 55). This is reflected in the differentiation between “developmental” and “acquired” pedophilia in the literature where acquired pedophilia is etiologically associated with a structural brain abnormality and developmental pedophilia is not (54, 56). However, the diagnostic manuals do not differentiate between these two types of pedophilia, as the diagnoses are symptom-based and do not consider etiology.

Also for antisocial behavior, there are associations between damage of the prefrontal cortex, be it due to a head injury or due to neurodegeneration like in Frontotemporal Dementia, and the occurrence of antisocial behavior in previously normal people (57). Cases of severe ventromedial prefrontal lobe epilepsy have been described that were associated with persistent antisocial behavior that was reversible after epilepsy surgery (58). In these cases, abnormal behavior is associated with a brain pathology which suggests a causal link between this pathology and the deviant behavior.

On the other hand, someone can behave in the same way for completely different reasons. For example, someone could live in a subculture where it is normal to behave in an antisocial or even criminal way to be “successful”. If it is normal in the social environment to make a living from, for example, drug dealing or criminal financial transactions, it could be reasonable to follow this tradition. Another example is someone who shows hypersexual behavior because he simply has no reason to confine himself due to money and power. In these cases, there is no reason to assume an underlying pathology. It is rather a morally questionable behavior.

The point here is: the fact that there are cases of brain pathologies leading to disinhibited or antisocial behavior doesn’t imply that all people behaving in the same way have a brain pathology.

### Wakefield’s “Harmful Dysfunction” Model

The question of the underlying dysfunction in ASPD and Pedophilic Disorder seems to be crucial for defending their status as mental disorders. A frequently cited concept related to the DSM definition of mental disorder is Wakefield’s “harmful dysfunction” model (4). This model assumes that a mental condition can be classified as a mental disorder when two criteria apply: Firstly, it is the result of a dysfunction, understood in an evolutionary sense as the failure of a process to perform the function it was biologically designed for; secondly, it is harmful to the individual according to sociocultural standards (4). By this definition, Wakefield tries to escape definitional problems by combining, as he calls it, a “value term” (harm) and a “scientific and factual” term (dysfunction) (4). The idea is to evade two problems: On the one hand, a mere “scientific” concept of mental disorder leads to the problem that every deviation from a scientifically defined standard could be viewed as a mental disorder even though the affected individual is neither suffering nor impaired. On the other hand, a mere value-based concept of mental disorders entails the risk of pathologizing socially disvalued behavior. Thus, according to Wakefield, only a harmful dysfunction represents a mental disorder, not a dysfunction without any harm to the individual nor something evaluated as harmful (according to sociocultural standards) but without representing a dysfunction.

We will come back to the notion of dysfunction in Pedophilic Disorder and ASPD later. Regarding the harm criterion, ASPD and Pedophilic Disorder are special since most mental disorders are primarily harmful to the affected individual. For “vice-laden” disorders like ASPD and Pedophilic Disorder, however, the “harm-criterion” primarily concerns others. Of course, some persons with Pedophilic Disorder might experience personal distress, probably after having internalized the society’s negative attitude towards pedophilia. Some persons with ASPD, however, may even enjoy real benefits through their special personality traits, both in terms of income and reproductive success. Malon (11) introduces the concept of “dangerous dysfunction” instead of “harmful dysfunction” in the case of Pedophilic Disorder, arguing that it is actually the concept of “dangerous dysfunction” that explains the presence of Pedophilic Disorder in DSM.

### Alternative Definitions of Mental Disorder

In the diagnoses of ASPD and Pedophilic Disorder, harm to the individual in the sense of personal distress or impairment is not necessarily implied. However, harm to the individual might be present even without the person concerned being aware of it. The philosopher Graham (59) states that having a mental disorder does not necessarily comprise the recognition of its harmfulness by the affected individual herself. According to Graham, a mental disorder is a disability, dysfunction or impairment in one or more basic mental or psychological faculties or capacities of a person that has harmful or potentially harmful consequences for the person concerned (59) (p. 28). It is a disorder because it is harmful in the sense that the person is worse off with the disorder than without the disorder, that she cannot control it, and that it cannot be removed by using additional psychological resources, *e.g.* by simply “pulling oneself together”.

Insofar, a person with Pedophilic Disorder could be regarded as worse off with the disorder than without it because having it means that either he has to abstain from fulfilling sexual relationships his whole life or he will commit a criminal act and possibly be punished for it. However, this argument is valid only for pedophilic persons living in societies which condemn and regularly punish child sexual abuse. In the case of ASPD, one could argue that the person is worse off with the disorder than without it because he is, for example, not able to have good relationships with other people. This, however, presupposes a certain model of good relationships and a “good life”, and therefore is value-laden and moralistic.

Heinz et al. (60, 61) argue for a differentiation between mental diseases in a narrow sense and states of suffering or disorders in a broader sense that do not meet the criteria of a disease. This differentiation, however, is not made by DSM and ICD where the notion of mental disorder is used for all diagnoses. Heinz et al. demand that the notion of mental disease should only be applied when life-relevant functional abilities are impaired and the affected person suffers from it or is impaired in her ability to cope with everyday life. Applying such a standard, many currently classifiable disorders are not diseases in this sense (60, 61). However, they are more or less easily classifiable states of suffering for which psychotherapeutic help and possibly drugs can be offered (60, 61). In this sense, Pedophilic Disorder and ASPD are not mental diseases.

### What is a Mental Dysfunction?

The concept of mental dysfunction is central in most definitions of mental disorder. However, there is no consistent definition of this concept. For example, DSM-5 uses the notion of dysfunction without elucidating it.

Schramme (62) distinguishes four models of mental functions. The first model, for which Wakefield’s concept of dysfunction is the most prominent example, is based on evolutionary psychology. According to Wakefield, mental functions result from selection processes and thus enable individuals to solve problems of adaptation (4). Schramme rightly criticizes the historical orientation of this theory: Some processes may have been adaptive to past environments but not to our present environment. The second model of mental functions comes from cognitive psychology. Functions in this sense are best understood in formal terms as “input–output-relations”, not in any teleological sense. Schramme notes that this theory hardly applies to the concept of mental disorder, because it does not imply “normativity”, that means, it has no concept of how a mental function should work, and thus no concept of dysfunction. The third model supports a goal theory of function and is close to Boorse’s disease theory that identifies survival and reproduction as the highest goals of organisms (8). Mental functions are thus understood through their relation to these goals. In contrast to evolutionary psychology, this model does not refer to the evolutionary selection of these functions but evaluates them with regard to the present environment. Schramme, however, criticizes that this model lacks a plausible model of the “psychological species design” with regard to survival and reproduction. The fourth model is the ‘value-theory’, for which there is no established psychological account. This model determines functions according to their contribution to human welfare and the good human life. A mental function thus allows for the individual to live a good life. However, such a theory is always at risk of confounding a certain way of life with mental health.

## Discussing the Disorder Status of Pedophilic Disorder and ASPD

As we have argued, in both the definitions of ASPD and Pedophilic Disorder behavior harmful to others or even criminal behavior is a criterion for the diagnosis of a mental disorder.

If we thus conclude that ASPD and Pedophilic Disorder are just a “medicalization” of vice conditions, we have to ask whether and, if so, how these diagnoses can still be justified within a medical model.

### Neurobiological Findings in Pedophilic Disorder and ASPD

The most influential argument to justify the diagnoses of ASPD and Pedophilic Disorder within a medical model seems to be a “conservative” one. These diagnoses are well established, they have a long clinical tradition and some prognostic utility (18). This supports the argument that they should only be changed if there is strong empirical evidence that another nosological construct is more valid than the established ones.

The idea of a validation of the existing nosological constructs is pursued by researchers investigating underlying neurobiological and neuropsychological alterations in persons with ASPD or Pedophilic Disorder. There is a growing body of research indicating that there might be deviations in the brains of persons with ASPD and Pedophilic Disorder. However, the interpretation of these findings needs to be handled with care: Are the neurobiological deviations a sign of a pathology, or a sign of a vulnerability, or a consequence of a disease, or only a normal variant? And further, can these neurobiological differences causally explain the behavior (at least partly)?

For ASPD, studies show structural and functional deviations mainly in the areas of the amygdala, the striatum and the prefrontal cortex (43, 57, 63). Genetic etiological studies suggest an association of a gene x environment interaction of MAOA enzyme deficiency and childhood maltreatment with antisocial behavior (57, 63). Evidence for developmental factors in the etiology of ASPD comes from studies that suggest a link between prenatal factors, such as birth complications, maternal smoking and alcohol consumption during pregnancy, or prenatal nutritional deficiency, and the occurrence of antisocial and violent behavior (57, 64). Also, an association between maltreatment during childhood and maternal withdrawal in infancy and ASPD has been found (64). These findings suggest, that biological and social factors play a role in the development of ASPD, while “the presence of both factors exponentially increases the rates of antisocial and violent behavior” (64) (p. 4).

For Pedophilic Disorder, reduced amygdala volumes were found in several studies (65, 66). The association between pedophilia and increased rates of left-handedness, more head injuries before age thirteen, and lower intelligence suggest that neurodevelopmental factors play a role in the development of pedophilia (66). These findings support, though do not prove, the idea of underlying neurobiological alterations in Pedophilic Disorder.

However, most of the studies have severe methodological flaws.

For Pedophilic Disorder, most of the studies show a sampling bias in investigating only incarcerated pedophilic child sexual offenders with very scant evidence on non-offending pedophiles (65, 66). It is thus not clear whether alterations found in the brains of pedophilic child sexual offenders are causally contributing to their pedophilic preference itself or whether they are rather associated with offending in general by, for example, contributing to diminished behavioral control or lower intelligence. The latter assumption is supported by a MRT study by Schiffer et al. (67), which provided first evidence that child sexual offending in pedophilia rather than pedophilia alone is associated with structural brain differences. Their study was published in the context of the German multi-sided research network NeMUP that investigated differences between pedophilic and non-pedophilic men, between child sexual offenders and non-offenders, and between convicted and non-convicted (pedophilic) child offenders.^14^

In the case of ASPD, the main methodological problem seems to be confounding variables, since most of the persons with ASPD show psychiatric comorbidities like substance use disorder or mood disorders (43). Another problem is the questionable homogeneity of persons that fulfill the criteria of ASPD. A study by Gregory et al. (71), for example, found significant differences in gray matter volume in the prefrontal cortex between offenders with ASPD and additional psychopathic traits and offenders with ASPD without psychopathic traits, but not between offenders with ASPD without psychopathic traits and non-offenders.

These findings show the need for better study designs to get more reliable results. However, even if we get better results, we still face the general problem of interpreting neurobiological differences as indicated above. The finding of a neurobiological difference is not equivalent to a dysfunction, understood in psychological terms. The question of dysfunction is superior to it. An atypical structure or function of the amygdala, for example, is not per se dysfunctional or pathological. The assessment of its dysfunctionality depends on its assumed effects on the psychological and behavioral level, and how these effects are evaluated. An atypical function of the amygdala could even be evaluated as advantageous because it is associated with less anxiety.

### Dysfunction in Pedophilic Disorder and ASPD

A crucial point in any discussion about the disorder status of a mental condition is the question if there is a convincing model of dysfunction, understood in psychological terms.

#### Pedophilic Disorder

With regard to pedophilia, one could argue under an evolutionary account of dysfunction, that it is a form of a sexual dysfunction, assuming that the biologically defined function of sexual arousal (*i.e.* the reason the mechanism of sexual arousal was selected for) lies in its contribution to (potential) reproduction (72) (p. 499), which is clearly not the case in pedophilic sexual behavior. This, however, is an insufficient model of the function of human sexuality. Human sexuality has important functions beyond reproduction, particularly promoting pair bonding and fulfilling emotional needs. Many forms of sexuality that do not pursue reproduction are broadly accepted, *e.g.* sexual intercourse of infertile people, under birth control, or homosexuality. Furthermore, there is no reason not to use a certain function for other, possibly purely hedonistic purposes that have nothing to do with its evolutionary function. The fact that a function is used for other than the alleged evolutionary purposes does not mean that this is dysfunctional.

Some pedophilic men actually state that they are not only interested in sexual contact with children but also look for romantic relationships with them (73). The dysfunction in Pedophilic Disorder thus cannot simply stem from the fact that the sexual arousal is not associated with (potential) reproduction. The concept of a dysfunction in an evolutionary sense falls too short here.

According to DSM-5 and ICD-11, a pedophilic sexual interest is only deemed a mental disorder when it leads to subjective distress or impairment, or has been acted upon.

To assume that having certain sexual fantasies or urges is not pathological but acting according to them is, seems inconsistent. It might be explained by the implicit assumption that there is another dysfunction involved, namely an impaired ability to control one’s behavior. To illustrate this point: if a heterosexual teleiophilic man (*i.e.* a man sexually attracted to physically mature individuals) sexually assaults a woman, it is not generally supposed that he must be mentally disordered because he couldn’t control his sexual urges. For it is just as possible that he thought the assault was justified, *e.g.* because the woman dressed “lewdly”. There is no reason to regard the case of the heterosexual teleiophilic sexual offender differently from the case of a pedophilic sexual offender who is convinced that his behavior is morally justified, or who just does not respect the rights of children.

Moser (74) rightly argues that a diagnosis of a paraphilia does not imply a lack of the ability to control one’s behavior: “Those individuals who cannot control their sexual impulses may qualify for another diagnosis based upon their inability to control their impulses, but not based upon the specific sexual behavior.” (p. 323).

This analysis shows that a model of dysfunction measured by moral standards is employed for Pedophilic Disorder. This argument is supported by the fact that the appraisal of sexual activities with children depends on historical and cultural contexts and has been accepted at varying times and cultures (22). This, of course, does not morally justify sexual acts with children. Only cultural relativists would conclude that sexual acts involving children are morally permissible because they are accepted in some cultures. We, however, regard child sexual abuse as a violation of universal human rights, including children’s rights. Thus, the fact that child sexual abuse is not sanctioned in some countries is no valid argument against its moral wrongness and its legal prohibition.

#### ASPD

In the case of ASPD, one could argue that antisocial behavior represents a dysfunction in social functioning. This argument implicitly presupposes that prosocial behavior is normal human behavior. However, under an evolutionary account, in many or even most societies during human history antisocial behavior was probably “adaptive” because it was the “normal and efficient” way to success, both in terms of reproduction and material wealth. Only in civilized societies governed by the rule of law, antisocial behavior becomes less adaptive than prosocial behavior and is considered abnormal and dysfunctional.

Some authors suggested that psychopathy could also be understood in evolutionary terms due to frequency-based selection as “adaptive” behavior (49, 75). According to this idea, a society with a prosocial majority can tolerate a small number of psychopaths that pursue their goals without being restrained by “other-regarding norms”. Reimer (49) argues that the typical personality traits of psychopaths, like experiencing less anxiety and being able to resist attempts of “moral” social reinforcing, can also be understood as advantageous under a pro-individualist account of human existence. Maibom argues that psychopathy is not a disorder at all, but “from a certain perspective, what we call deficits are actually advantages” (75) (p. 34).

## Practical Arguments for Considering Pedophilic Disorder and ASPD as Mental Disorders

Classifying something as a mental disorder is not only a theoretical question, but also has practical implications that need to be considered.

Most persons with Pedophilic Disorder and ASPD don’t seek help (11, 43). For ASPD, individuals presumably often don’t feel pain and thus have no motivation to change their condition (46). For Pedophilic Disorder, the possible reasons for not seeking help range from not feeling distressed by it, or not recognizing its potential harmfulness towards others to a lack of knowledge about possibilities to get help and shame and fear of stigmatization (76).

However, as the study of Levenson et al. (76) also shows, some persons with Pedophilic Disorder are willing to get help. As an example, the Dunkelfeld (“dark field”) project in Berlin, Germany, a voluntary prevention project for pedophilic men at risk of offending, shows that a significant number of pedophiles seeks help (77).

In many countries, the diagnosis of a mental disorder justifies treatment within the publicly funded health system. For that reason, the diagnoses of ASPD and Pedophilic Disorder can serve a useful purpose for individuals who feel distressed by their condition. If the health system with its long clinical experience can offer help, then it should do so (72).

However, the question is whether we need the diagnoses of Pedophilic Disorder and ASPD so that these persons can get help. For social problems social institutions outside the health system could be conceivable that offer help. Even if these diagnoses were removed from the diagnostic manuals, people could get help within the health system for comorbid conditions like depression or anxiety disorder if these mainly cause their personal distress. In the case of paraphilias, Moser et al. argue that “other psychological characteristics describe these individuals and their concerns more accurately than their sexual interests do” (20). Indeed, 93% of a sample of pedophilic sex offenders showed psychiatric comorbidities, mostly mood and anxiety disorders and substance use disorders (78). ASPD is also associated with anxiety disorders and substance use disorders. For the latter a prevalence of 80–85% among persons with ASPD was reported (43).

One could object that these comorbidities possibly are a consequence of the Pedophilic Disorder or ASPD and therefore the focus of treatment should be the Pedophilic Disorder or ASPD as the primary condition. However, the fact that there are almost no effective treatments for Pedophilic Disorder or ASPD yet indicates that what actually can be treated within the health system might rather be associated disorders like depression, anxiety, or substance use disorder and not ASPD or Pedophilic Disorder itself.

Both, ASPD and Pedophilic Disorder, are supposed to be associated, besides others, with neurodevelopmental factors (57, 66), which makes it difficult to therapeutically intervene as late as in adulthood. The goal of therapies is thus rather the prevention of future deviant behavior in order to avoid harm to others. As Seto (79) puts it regarding Pedophilic Disorder: “Instead of a ‘cure’, the focus of treatments for nonoffending individuals with pedophilia or hebephilia is the development of more effective self-management, to prevent sexual offending.” (p. 209).

The idea of drug treatment with antiandrogens or GnRH analogs (androgen deprivation therapy, ADT) in Pedophilic Disorder is not to change sexual preference but to reduce sex drive and thereby reduce the risk of (re-)offending. There is, until now, very limited evidence of the efficacy of ADT, and the level of willingness to undergo this kind of treatment is quite low (79, 80). Furthermore, according to a review of studies on behavioral and cognitive–behavioral treatments of pedophilia, there is no reliable evidence of their long-term efficacy (23). There are, however, few hints that it might be possible to actually modify sexual interest in children by, for example, strengthening self-esteem, coping skills, emotional self-regulation, and relationship skills in order to enable men with a sexual interest in children to fulfill their emotional and sexual needs with adult partners (81). Studies on specific techniques, like masturbatory reconditioning in order to suppress deviant sexual interests and/or enhance normative sexual interests, show scant evidence of their efficacy to date (82).

For ASPD, a meta-analysis by Wilson (83) shows no significant effects of treatments. A lack of high-quality studies and small sample sizes might contribute to these findings. Better designed studies with larger sample sizes are required for future research.

It seems necessary to classify ASPD and Pedophilic Disorder as mental disorders in order to facilitate further research on them, gain better insights into their etiology, and develop new therapies. The example of the “psychopathy”-concept, however, shows that there can be a lot of research on a concept without being an official diagnosis in DSM and ICD (44). The psychopathy-checklist (PCL-R) is widely used in forensic contexts to reliably assess the risk potential of criminals with psychopathic traits (24). Since psychopathy does not need to be a diagnosis in DSM and ICD to be a broadly applied concept, it seems that ASPD and Pedophilic Disorder do not need it either.

Similar to psychopathy, ASPD and Pedophilic Disorder are most relevant in forensic contexts (25, 38). Apart from clinical utility, the forensic implications of these diagnoses need to be considered. According to Sexually Violent Predator laws in many U.S. states, sex offenders with a “mental abnormality” and a high risk of re-offending can be indefinitely committed after the prison sentence to protect society from them (84). Even though “mental abnormality” is a legal term referring to an impairment in emotional and volitional capacity that predisposes to the commission of criminal sexual acts and not synonymous with “mental disorder” (85), the diagnosis of a paraphilic disorder, as specified in DSM, is practically mostly accepted as sufficient to ascertain “mental abnormality” (86). Regarding these severe consequences, the definition of the paraphilic disorders in DSM seems especially critical.

## Conclusions

### “Vice-Laden Disorders” in Psychiatry

Diagnoses that primarily rely on behavior harmful to others, like Pedophilic Disorder and ASPD, fall out of the general disease concept. They even do not meet the general criteria of mental disorders as defined by DSM-5 or the “harmful dysfunction” model by Wakefield. Neither the criterion of harm to the individual himself, nor the criterion of a dysfunction are met in these two diagnoses.^15^ Instead, they rely on another disease criterion: the criterion of harm to others. Psychiatry brings itself into great conceptual difficulties by making behavior harmful to others/criminal behavior a central part of the definition of some mental disorders, while at the same time lacking a clear concept of dysfunction in these cases. When diagnoses are formulated in a way that makes it possible to apply them to mere antisocial and criminal behavior, psychiatry is at risk of confounding the medical and the moral.

Furthermore, the purely behavioral diagnoses do not reveal whether the behavior is based on a mental dysfunction or whether it was chosen voluntarily or for specific reasons.

Therefore, the formulation of the criteria sets of “vice-laden” disorders needs to be done very cautiously in order to avoid a confusion between criminal/immoral behavior and mental disorder. It should not be possible that harming others/criminal behavior defines a mental disorder. A psychiatric diagnosis should not only rely on observable behavior, but consider psychological, cognitive, or affective factors as well.

After considering the arguments for and against the disorder-status of Pedophilic Disorder and ASPD, we come to different conclusions regarding both diagnoses.

### The Disorder-Status of Pedophilic Disorder

In the case of Pedophilic Disorder, we think that the diagnosis should be kept but reformulated in accordance with the general definition of mental disorder in DSM-5 in order to make it consistent with a medical model of mental disorder. This means it should only be applicable to individuals that are distressed or impaired by it so that they can get treatment within the health system. It should not be possible to make the diagnosis solely based on behavior harmful to others. Therefore, we suggest reformulating Criterion B of Pedophilic Disorder as follows: “The sexual urges or fantasies cause marked distress or interpersonal difficulty (*e.g.* in the context of occupation, family life, friendships, intimate life).” That means, the criterion “The individual has acted on these sexual urges” is cancelled.

Our suggested reformulation of Criterion B is indeed consistent with the form it already had in DSM-IV. As De Block et al. (87) note, the DSM-IV diagnostic criteria were “by far the most consistent vis-à-vis the DSM’s own definition of mental disorder” (p. 291). It was, however, criticized that this criteria set leads to the situation that someone acting on his pedophilic interests without feeling distressed would not be considered mentally ill (88). O’Donohoe et al. (89) argue that rather the lack of experiencing subjective distress when being sexually attracted to children than the experience of distress is a sign of psychological problems. They do not accept that, according to DSM-IV, a “contended pedophile” does not meet the criteria of a mental disorder. They argue that a person sexually interested in children must be considered in some way socially impaired “because societal norms dictate that it is abnormal for a person to be sexually interested in children” (p. 102). They clearly want to classify pedophilia as a mental disorder for social and forensic rather than for medical reasons. Their postulation that “a single instance of sexual behavior with a child should be sufficient to label someone as having a disorder” (89) (p. 103) confounds criminal behavior with mental disorder.

If pedophilia by itself is not a mental disorder according to DSM-5, then acting according to it cannot be a mental disorder unless there is clear evidence of a dysfunction of volitional control. Impairment of volitional control, however, is not implied in the diagnosis of a paraphilic disorder (85). If we assume that sometimes such impairment is given, then it probably stems from another disease (like *e.g.* dementia, a brain tumor or mental retardation). If there is no such impairment, we have to assume that this person acted deliberately, and it is not clear why this should be a sign of a mental disorder rather than simply a criminal act.

The DSM-5 warns of the dangers of using a diagnostic manual developed for clinical purposes in the forensic context. For assigning mental disorder in the legal sense “additional information is usually required beyond that contained in the DSM-5 diagnosis, which might include information about the individual’s functional impairments and how these impairments affect the particular abilities in question” (3) (p. 25).

It is important to note that there is a difference between a mental disorder and the US-American legal concept of “mental abnormality”.

We suggest that it should be possible to diagnose a “mental abnormality” in the forensic sense for a person with pedophilia who is neither distressed nor impaired by his pedophilic condition (*i.e.*, who fulfills criterion A but not B according to our suggestion). Even though this person does not meet the criteria of a mental disorder as suggested by us, he might still meet the concept of “mental abnormality” if there is evidence of a high risk of reoffending. We thus suggest that this difference in clinical and forensic use is clearly annotated in the diagnostic criteria of Pedophilic Disorder in DSM. This suggestion is important with regard to other countries than the USA. The DSM is used worldwide for research, and therefore its diagnostic criteria should not be distorted in order to adapt them to the US legal system. In Germany, for example, no diagnosis of a mental disorder is required to order preventive detention after imprisonment; rather the assessment of danger and the prognosis of the probability of recidivism is decisive.

Our intention is not to protect the “contented pedophile”, as long as he is dangerous, from preventive detention or to downplay the harm that child molesters do to their victims in any sense. On the other hand, our suggestion is not meant to preclude the detained child molester from getting treatment if at some point he starts to show insight into his problems and wants to get treated. Rather, we want to separate the medical aspects of Pedophilic Disorder from the societal and forensic implications.

To summarize, our suggestion is as follows. We agree with the differentiation between Pedophilia and Pedophilic Disorder in DSM-5 and suggest adding a category “Pedophilia with mental abnormality” for forensic purposes. Thus, we suggest defining Pedophilia as pedophilic preference without distress/impairment; Pedophilic Disorder as pedophilic preference with distress/impairment; and Pedophilia with mental abnormality as pedophilic preference with sexual offending and high risk of re-offending with or without distress/impairment.

### The Disorder-Status of ASPD

In the case of ASPD, however, we think that the arguments to remove it as a distinct diagnosis from the diagnostic manuals are stronger than the ones to keep it. Especially the presumed lack of personal distress of individuals with ASPD and the strong correlation with criminal behavior and incarceration indicate that this diagnosis is more of a social than a mere health-related problem.

We agree with Kröber and Lau (15) who said: “If those with antisocial personalities, like anyone else, are subject to social influences and learning processes, they act as rational and competent citizens; their decision against behaving in compliance with standards should not be considered as pathologic.” (p. 687).

Herpertz and Sass (90) warn of the consequences of confounding antisocial behavior with “real” disorders in forensic psychiatry: “If the forensic psychiatrist fails to distinguish clearly between simple antisocial behaviour and a profound disturbance in personality, psychiatry runs the risk of being charged with handling all kinds of recurrent social deviance and delinquency. This would greatly hamper our capacity to treat those offenders who show real and treatable mental disorders.” (90).

As Gert & Culver (41) put it: “If psychiatry is to take its place as a branch of medicine, mental disorders, like physical disorders, should be limited to conditions that cause harm to the person with the disorder.” (p. 489).

We think that the implementation of a dimensional model of personality disorders, as introduced by ICD-11, will mitigate the problem of attributing a diagnosis of mental disorder to mere criminal behavior. The ICD-11 does not contain the diagnosis “Dissocial Personality Disorder” anymore. Antisocial or dissocial personality traits will then be a specifier among others in the diagnosis of a general personality disorder. Thus, with this new model, the focus will hopefully be more on the cognitive, affective and interpersonal dimensions of personality disorders while avoiding an overly focus on deviant behavior.

To summarize: We suggest removing ASPD from the DSM, and support the planned removal of the diagnosis DPD from the ICD-11.

### Practical Implications

Our suggestion to remove or reformulate the “vice-laden” diagnoses does not imply the demand for stopping research on them—quite the contrary. Especially in the forensic context, it is important to find opportunities to effectively prevent their harmful consequences and develop treatment methods insofar this is possible. The concept of psychopathy shows that an official diagnosis is not necessary for research to be done on forensically relevant conditions.

Regarding Pedophilic Disorder, our suggestions strongly support therapeutic offers (like the Dunkelfeld project) for people who feel distressed or impaired by their condition and seek help.

Regarding antisocial behavior, we think that it is much more of a social problem that has to be addressed more by other societal systems than the health system.

Finally, our suggestions have legal implications in some legal systems. Particularly for the USA, we suggest adding the category of “pedophilia with mental abnormality” in DSM for forensic use in order to separate the clinical and forensic aspects of pedophilia. However, the requirements of the legal systems in some countries are no valid argument against clear conceptual differentiations in the psychiatric diagnostic systems.

## Author Contributions

Development of the concept (SM, RM). Writing of the paper (RM). Literature research (RM, SM). Discussion of the concept (RM, SM, HW). Editing the text (SM, HW). All authors contributed to the article and approved the submitted version.

## Funding

ERA-NET NEURON and the Federal Ministry of Education and Research (BMBF) of Germany funded the work of SM (grant number: 01GP1621A). The research of RM and HW did not receive any specific grant from funding agencies in the public, commercial, or not-for profit sectors. We acknowledge support from the German Research Foundation (DFG) and Open Access Publication Fund of Charité—Universitätsmedizin Berlin.

## Conflict of Interest

The authors declare that the research was conducted in the absence of any commercial or financial relationships that could be construed as a potential conflict of interest.

## Footnotes

1 Contrary to medical mainstream opinion, Hucklenbroich regards asymptomatic carriers of infectious diseases as ill. According to his theory (see *The general concept of disease*), asymptomatic carriers fall within the scope of disease criterion 5 (2).

2 The rationale of our argumentation applies to other diagnoses as well, like for example “Coercive sexual sadism disorder” in ICD-11. We have chosen Pedophilic Disorder and ASPD because they are the most questioned and relevant diagnoses.

3 Charland (5) argues that only the personality disorders in Clusters A and C are genuine clinical disorders. In contrast, he considers the Cluster B disorders (which include antisocial, borderline, histrionic, and narcissistic personality disorder) as moral disorders since their definitions are “morally loaded” and they require “moral treatment”.

4 A psychiatric diagnosis *per se* is not a reason for assuming a lack of criminal responsibility or diminished responsibility but it is part of the forensic examination. According to German criminal law, “[a] person acts without guilt who, at the time the criminal act is committed, is incapable of understanding the wrongfulness of his or her action or is incapable of acting in accordance with this understanding due to mental illness, due to a profound disturbance of consciousness, or due to mental retardation or another serious mental abnormality” [Section 20, German Criminal Code, English translation cited from (15)]. Diminished responsibility is present in the case of a diminished capability of the offender to understand the wrongfulness of an action or to act in accordance with this understanding due to one of the reasons indicated in Section 20 and may lead to mitigated penalty (Section 21, German Criminal Code). Section 20 lists four mental conditions that are necessary prerequisites for assuming a lack of criminal responsibility. However, these mental conditions are not equivalent to specific psychiatric diagnoses. They are legal terms that refer to psychiatric diagnoses (16).

5 The general concept of the personality disorders has been criticized fundamentally. Lieb criticizes the concept of personality disorder as contradictory in itself and as harmful to the patient and to the therapeutic relationship (21).

6 It was proposed to include the attraction to pubescent children and rename the diagnosis “pedohebephilic disorder”, to include a victim count and the use of child pornography in criterion B, and to include the specifiers “in remission” and “in controlled environment” (31). After the refusal of these changes, it was criticized that Pedophilic Disorder is the only Paraphilic Disorder in DSM-5 that lacks the specifiers “in full remission” and “in controlled environment” (32, 33). Further criticism was directed against the refusal to include the attraction to pubescent children (27). These discussions, however, are not in the focus of this paper.

7 Data on the proportion of pedophilic and non-pedophilic child sexual offenders are quite scarce and come from small studies. According to Seto et al. (37), in a sample of 100 child pornography offenders (where the authors assumed a high probability of pedophilic interest due to phallometric responses), 57% were not known to have had sexual contact with children. Conversely, the prevalence of pedophilic preference among identified child sexual offenders is estimated at about 40–50% (based on their sexual arousal to stimuli depicting children or their sexual offense history) (23). First (38) notes that “compared with other paraphilic disorders, child molestation is even more likely to occur for nonparaphilic reasons”. Nonparaphilic reasons may be “a lack of more preferred sexual opportunities, hypersexuality, indiscriminate sexual interests, or disinhibition as a result of substance use or other factors” (23) (p. 393). Knack et al. (39) name “a general anti-social orientation”, “a sexual interest in coercion”, “attitudes accepting of sex between adults and children”, and “indiscriminate or opportunistic sexual behaviours” as reasons for non-pedophilic child sexual abuse (p. 183). Strassberg et al. found that non-pedophilic child molesters are more likely to show psychopathic traits than pedophilic child molesters (40).

8 ASPD and psychopathy are largely overlapping concepts. According to Ogloff (25), 81% of persons diagnosed with psychopathy also meet the criteria of ASPD, whereas only 38% of the persons with ASPD receive a diagnosis of psychopathy. This indicates that the population of persons diagnosed with psychopathy can more or less be considered a subset of the population of persons diagnosed with ASPD. Exceptions are typically fraudulent personalities (or so-called “white collar offenders”) who are psychopaths but do not meet the criteria of dissocial or antisocial personality disorder (45).

9 For the sake of clarity, we will mainly refer to Antisocial Personality Disorder in this paper, even though many of the points made equally apply to Dissocial Personality Disorder. However, because of the stronger focus on behavior in ASPD compared with Dissocial Personality Disorder, we consider the diagnosis of ASPD as more problematic.

10 Translated by Sabine Müller.

11 The alternative model for personality disorders in DSM-5 has been developed for further research (Section III). In the alternative model, personality disorders are generally characterized by impairments in personality functioning (Criterion A) and pathological personality traits (Criterion B). Personality functioning (Criterion A) involves self-functioning (identity and self-direction) and interpersonal functioning (empathy and intimacy). For each of these four elements, five levels of impairment (ranging from no impairment to extreme impairment) can be differentiated. Pathological personality traits (Criterion B) are organized in five broad domains, namely negative affectivity, detachment, antagonism, disinhibition, and psychotism. The impairments in personality functioning and personality trait expression are relatively inflexible and pervasive across a broad range of personal and social situations (Criterion C). They are relatively stable with onset in at least adolescence or early adulthood (Criterion D), cannot be better explained by another mental disorder (Criterion E), are not attributable to the physiological effects of a substance or another medical condition (Criterion F), and not better understood as normal for an individual’s developmental stage or sociocultural environment (Criterion G) (3) (pp. 761–3).

12 As soon as a crime is committed against an individual person, the perpetrator comes into conflict not only with the victim but also with the society whose moral or legal norms have been violated.

13 As an example, in Germany there were fierce debates about the harmfulness of sexual interactions between adults and children in the 1960s to the 1980s. Some sexologists, psychologists, and psychiatrists denied the harmfulness of sexual interactions with children (52). Pedophilic activists demanded the abolition of the legal age limit of sexual interactions, a position that was supported even by several leaders of the Green party (53).

14 The NeMUP researchers found that executive dysfunctions are related to offense status rather than pedophilic preference (68). Furthermore, they revealed that offenders and non-offenders differed in age, intelligence, educational level and experience of childhood sexual abuse, whereas pedophiles and non-pedophiles mainly differed in sexual characteristics (*e.g.*, additional paraphilias) (69). When they compared convicted and non-convicted pedophilic child sexual offenders, they found only two significant differences between the two groups. The convicted offenders had a higher interest in prepubescent children and had committed significantly more sexual offenses against children compared to non-convicted subjects (70). However, significant differences regarding clinical characteristics, inhibition performances, neuronal activation, empathy and impulsiveness between the two groups were not found (70).

15 However, this conclusion is not equally applicable to definitions of mental disorder that do not require that the individual recognizes the harmful consequences of his condition, like the definition of Graham (59).
